# Descending necrotizing Mediastinitis caused by *Kocuria rosea*: a case report

**DOI:** 10.1186/1471-2334-13-475

**Published:** 2013-10-11

**Authors:** Mi Kyung Lee, Soon Ho Choi, Dae Woong Ryu

**Affiliations:** 1 Department of Thoracic and Cardiovascular Surgery, College of Medicine, Wonkwang University, Shinyong-dong 344-2, Iksan, Jeonbuk, Republic of Korea

**Keywords:** *Kocuria rosea*, Descending necrotizing mediastinitis

## Abstract

**Background:**

*Kocuria* species are gram-positive, non-pathogenic commensals. However, in immunocompromised patients such as transplant recipients, cancer patients, or patients with chronic medical conditions, they can cause opportunistic infections.

**Case presentation:**

We report the first case of descending necrotizing mediastinitis in a 58-year-old, relatively healthy woman caused by *Kocuria rosea*.

**Conclusion:**

Descending necrotizing mediastinitis due to *Kocuria rosea* can be successfully treated with prompt surgical drainage combined with antimicrobial therapy.

## Background

Descending necrotizing mediastinitis (DNM) is an acute form of mediastinitis caused by odontogenic or deep cervical infections such as tonsillitis and pharyngitis that descend into the mediastinum and pleural space through the cervical fascial planes [1]. DNM is a polymicrobial infection with predominance of both aerobic and anaerobic organisms [2].

Only a limited number of *Kocuria* infections are mentioned in literature. Furthermore, to our knowledge, this is the first case reported in the English literature of *Kocuria rosea* associated with DNM.

*Kocuria rosea* is an aerobic, gram-positive coccus that is generally considered as a non-pathogenic commensal that colonizes the oropharynx, skin, and mucosa. Nonetheless, it can cause an opportunistic infection in immunocompromised patients [3]. We report here the first case of DNM associated with *K. rosea* in a relatively healthy woman.

## Case presentation

**Figure 1:**
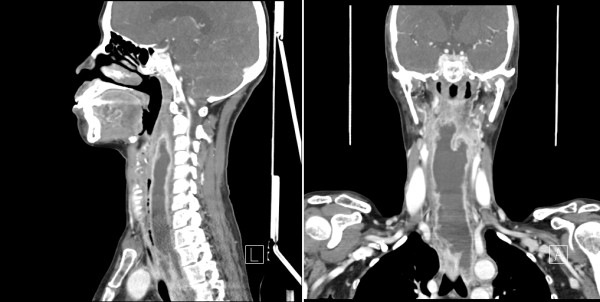
A computed tomography (CT) scan showing a large extended retropharyngeal abscess formation extending to the bronchial bifurcation.

**Figure 2:**
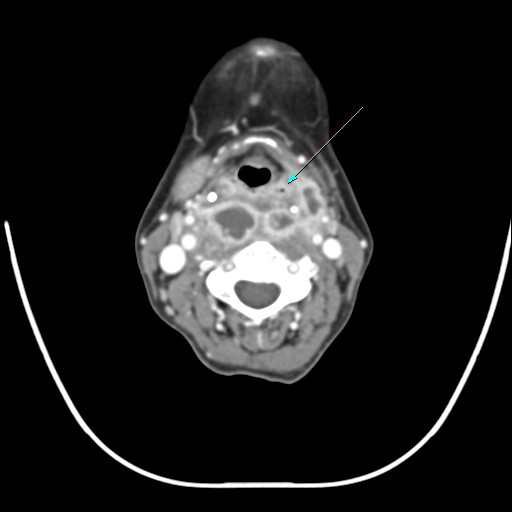
CT scan showing abscess formation beginning around the piriform sinus.

A 58-year-old woman presented to her local hospital with fever, myalgia, and sore throat. Her medical history was significant for gout and hypertension controlled with medications. After a few days of treatment, although her condition improved, she still complained of nausea, neck discomfort, and difficulty swallowing. Endoscopy revealed a gastric ulcer but no esophageal lesions. Ultrasound showed fluid collection in the neck space and a diagnosis of DNM was made by cervicothoracic computed tomographic (CT) scan. At that point, she was transferred to our hospital. On admission, she was afebrile with swelling of the neck and associated discomfort. She denied any other specific symptoms. Laboratory testing showed elevated levels of erythrocyte sedimentation rate (120 mm/h) and C-reactive protein (75.77 mg/L). The albumin level had decreased (3.2 g/dL) and she had a normal white blood cell count. The remaining laboratory values were within normal limits. A CT scan showed a large, retropharyngeal abscess extending from the surrounding piriform sinus to the bronchial bifurcation. No significant abnormality was seen in the pharynx and tonsils (Figures 1, 2).

After pre-operative systemic administration of a third-generation cephalosporin and clindamycin, the patient was promptly taken for emergency surgery. The mediastinum was approached using a thoracoscope, and the abscess was drained and irrigated with a Betadine solution mixed with saline. One chest tube was inserted into the neck through the abscess tract and another one was placed in the thorax for abscess drainage. Cervical drainage and irrigation were then performed in order to drain the remaining neck abscess.

A repeat CT scan obtained one day post-operatively showed a small residual central necrotic lesion in the left paratracheal area that was no longer evident on follow-up CT scan performed one week later. Using the Biomerieux Vitek 2 system, we isolated *K. rosea* from the exudate drained from the mediastinal abscess. The patient was discharged after 16 days of antibiotic therapy consisting of a third-generation cephalosporin and clindamycin. She has been followed up for 6 months and remains asymptomatic till date.

### Microbiological diagnosis

Culture of the abscess was performed with sheep blood agar, MacConkey agar, and thioglycollate broth. The plates were incubated at 35°C for 48 h. Anaerobic culture was performed using chocolate (Reduced) agar with an anaerobic pouch and thioglycollate broth and incubated at 35°C for 5 days. Anaerobic culture did not yield any microorganisms. The culture was positive for gram positive cocci arranged in tetrads; these cocci were non-hemolytic, catalase positive, coagulase negative, and nonmotile*.* Identification was performed using the Biomerieux Vitek 2 system(GPI card); however, the alternative means of identification, 16s rRNA sequencing was not performed. Additionally, antibiotic sensitivity tests were not performed.

## Discussion

Descending necrotizing mediastinitis is caused by a deep cervical or oropharyngeal infection that descends into the mediastinum and pleural space through several cervical fascial planes such as the carotid, prevertebral, retropharyngeal, and retrovisceral spaces. Among these, the retrovisceral space is the most vulnerable pathway leading to the mediastinum [1,2,4,5].

The primary origin of a deep cervical infection is mostly unknown, although it can be caused by an odontogenic infection, acute tonsillitis, a peritonsillar abscess, cervical lymphadenitis, sinusitis, or cervical trauma [4,5]. Although *Streptococcus* has been reported to be the most common pathogen responsible for DNM, it is usually a polymicrobial infection involving both anaerobic and aerobic organisms [4,6]. In a review of the English literature, Freeman et al. [2] found 96 patients with DNM between 1970 and 1999. All but 4 (4%) had mixed aerobic and anaerobic infections; in these 4 exceptions, the sole pathogen was beta-hemolytic *Streptococcus*.

The main predisposing factor complicating DNM due to a deep neck infection is multiple space involvement [7]. In addition, many reports have shown that coexisting morbidities such as diabetes mellitus, alcoholism, and chronic renal failure can predispose to the spreading of a deep neck infection into the mediastinum [2,4,5]. In spite of advances in antimicrobial therapy and increased use of CT scan as an early diagnostic tool, the mortality rate for this type of mediastinitis remains high and has been reported to be between 25 and 40%. This poor prognosis could be a result of diagnostic delay and inadequate surgical drainage [1,2,5,8]. Therefore, while early diagnosis and initial intravenous broad spectrum antibiotic therapy is important, surgical drainage and debridement of necrotic tissue is essential.

*Kocuria spp*. was classified as *Micrococcus spp*., then later reclassified in the new genus *Kocuria spp.* (*K. rosea; K. kristinae; K. varians; K. palustris; and K. rhizophila ap. Nov.*) by Stackebrant and colleagues [9]. Our literature review has found that *K. kristinae* is the most pathogenic organism of the *Kocuria spp.*[3,10-12] and coincidentally possesses some degree of facultative anaerobic characteristics unlike the other, strictly aerobic, *Kocuria* species [9,11]. The *Kocuria spp*. has been most commonly responsible for infections in chronically ill or immunocompromised patients. Only limited cases have been reported where this organism has caused infection in an immunocompetent subject [3,10-13]. To our knowledge, there are only 3 cases in the English literature in which *K. rosea* has been reported as a pathogen in infective endocarditis in an immunocompetent patient [13] and in catheter-related bacteremia [14] and, peritonitis [15] in immunocompromised patients. DNM caused by *K. rosea* in an immunocompetent host has not yet been reported making this the first such reported case.

Misidentification of coagulase-negative staphylococcus as *Kocuria* by using the VItek 2 system with ID- GPC card has been reported [16]. However, a new Vitek 2 gram-positive identification GP card allows for the identification of additional taxa and is reliable for identification of *Kocuria*[17]. Although the 16s rRNA sequencing may be used to confirm species identity, we, unfortunately, could not performed the analysis.

Because of the normal flora of the oropharynx and skin and the pattern of the abscess formation in this patient, we can assume that the abscess originated from an infection of the piriform sinus (Figure 2). Although we did not perform antibiotic susceptibility testing, the organisms were susceptible to many commonly used antibiotics in other reports [3,10-12,14,15]. Stackebrandt et al. [9] reported that all strains of *K. rosea* were susceptible to tetracycline, erythromycin, oleandomycin, novobiocin, methicillin, kanamycin, polymyxin, vancomycin, penicillin G, streptomycin, chloramphenicol, and neomycin. In our case, a third-generation cephalosporin and clindamycin were administered empirically prior to emergency surgical drainage. The same antibiotic regimen was then administered for 2 more weeks, after which time, the patient was discharged.

## Conclusion

*Kocuria rosea* comprises the normal flora in the oropharynx, skin, and mucosa. It generally causes infections only in immunocompromised patients. However, it can also be a causative pathogen of oropharyngeal and deep cervical infections in immunocompetent patients.

Although, *K. rosea* has a low pathogenicity and high susceptibility to a variety of antibiotics, prompt surgical drainage, debridement, and administration of broad spectrum antibiotics could show an excellent result in DNM caused by *K. rosea*.

### Consent

Written informed consent was obtained from the patient for publication of this case report and any accompanying images. A copy of the written consent is available for review by the Editor of this journal.

## Competing interests

The authors declare that they have no competing interests.

## Authors’ contributions

DR and SC performed the operation. ML carried out the clinical study of the patient. DR drafted the manuscript. All authors read and approved the final manuscript.

## Pre-publication history

The pre-publication history for this paper can be accessed here:

http://www.biomedcentral.com/1471-2334/13/475/prepub
